# Elevated nitric oxide and carbon monoxide concentration in nasal-paranasal sinus air as a diagnostic tool of migraine: a case – control study

**DOI:** 10.1186/s12883-021-02434-y

**Published:** 2021-10-26

**Authors:** S. M. R. Bandara, S. Samita, A. M. Kiridana, H. M. M. T. B. Herath

**Affiliations:** 1 Teaching Hospital Kandy, Kandy, Sri Lanka; 2 University of Peradeniya, Peradeniya, Sri Lanka; 3 National Hospital of Sri Lanka, Colombo, Sri Lanka

**Keywords:** Paranasal air suction, Acute migraine, Nitric oxide, Carbon monoxide

## Abstract

**Background:**

A recent study showed that 60–s paranasal air suction results in an immediate pain relief in acute migraine. This is the study to assess the Nitric Oxide (NO) and Carbon Monoxide (CO) concentration in nasal-paranasal sinus air of migraine patients and to compare it with healthy controls.

**Methodology:**

The NO and CO levels of air sucked out from nasal-paranasal sinuses of 20 migraine adolescent and young adults among school students, aged 16 –19 years, and 22 healthy similar aged school students as controls were measured as key responses using a portable NO and a portable CO analyzer.

**Results:**

Patients had comparatively high values compared to the controls for paranasal NO (both left and right sides), paranasal CO (both left and right sides), Fraction Exhaled NO (FeNO) and Fraction Exhaled CO (FeCO). Patients had median paranasal NO contents of 132.5 ppb and 154 ppb on left and right sides respectively compared to 36 ppb and 34.5 ppb corresponding values in controls (*P* <  0.0001). Similar pattern was observed with paranasal CO (*P* <  0.0001). FeNO and FeCO content were also higher in patients (*P* <  0.0001). Receiver characteristic operating curves of all gas measurements showed that they all could classify patients and controls effectively and NO was the most effective followed by paranasal CO. After air suction, the mean pain scores of general headache and tenderness dropped by a very large margin in migraine patients (*P* <  0.0001).

**Conclusions:**

Suctioned out high nasal-paranasal sinus NO and CO levels can be used to distinguish migraine patients from healthy subjects. In fact, suctioned out paranasal NO measurements of both sides with a cutoff point of 50 ppb provided a perfect classification of patients and controls. Increased sinus NO and CO during acute episode of migraine is an observation we had and we agree that further studies are needed to conclude that NO and CO can be a causative molecule for migraine headache.

**Trail registration:**

Clinical Trial Government Identification Number – 1548/2016.

Ethical Clearance Granted Institute – Medical Research Institute, Colombo, Sri Lanka (No 38/2016).

Sri Lanka Clinical Trial Registration number: SLCTR/ 2017/018 (29/06/2017).

Approval Granting Organization to use the device in the clinical trial– National Medicines Regulatory Authority Sri Lanka (16/06/2018), The device won award at Geneva international inventers exhibition in 2016 and President award in 2018 in Sri Lanka. It is a patented device in Sri Lanka and patent number was SLKP/1/18295.

All methods were carried out in accordance with CONSORT 2010 guidelines.

## Introduction

Migraine is a primary headache disorder that occurs at all ages and is characterized by recurring, moderate to severe headache that usually lasts from 4 h to 3 days with accompanying nausea, vomiting and sensitivity to light or sound. Sometimes it is preceded by an aura [1]. No causative molecule for migraine has been identified to explain the pathophysiology of migraine, even though many theories have been proposed [2]. Sinus Hypoxic Nitric Oxide theory (SHNOT) hypothesis for migraine states that Nitric Oxide (NO) may play an important role in migraine. In this hypothesis, diffused sinus nitric oxide (dsNO) in the nasal mucosa is suggested to be the main cause and the initiative molecule for migraine [3]. Inducible Nitric Oxide Synthetase (iNOS) is present in the epithelium, close to the ciliated surface of nasal and paranasal area, where nasal and sinus NO (sNO) is synthesized [4]. A large production of NO(s NO) takes place in the paranasal sinuses and is continuously released to the nasal air stream [5, 6]. The concentration of sNO may sometimes reach more than 20 ppm in a healthy sinus [7]. In addition, paranasal sinuses contributes to production of sinus Carbon Monoxide (s CO) too [8]. Hypoxia leads to increase production of NO and Carbon Monoxide (CO), which have vasodilatory and anti–inflammatory actions [9]. Administration of intranasal NO scavengers have been proven to neutralize nasal NO and reduce migraine attacks and the severity [10]. Suction of paranasal air mechanically can also be used to reduce NO production as well as NO stagnation within the nasal and paranasal cavities. Another similar study [11] showed that 60–s paranasal air suction results in an immediate pain relief in acute migraine headache and its other common symptoms such as photophobia, phonophobia, nausea, and generalized tiredness of the body, and the relief can last for more than 24-h period without any side effects.. It was assumed that the suction of air from the paranasal sinuses removes the neuro and vasoactive air molecules that could be the causative agents for migraine. However in these studies, the sucked out air has not been analyzed. Direct Assessment of paranasal sinus gases is costly and invasive. Therefore in this research NO and CO levels of the air sucked out from nasal-paranasal sinus were studied in migraine patients and normal healthy subjects as an indirect level of NO and CO in para nasal sinuses and were compared. This is the first case control study to assess the NO and CO concentration in paranasal sinus air of migraine patients.

## Methodology

This case control study was conducted according to STROBE statement guidelines. The participants were selected from two stage randomization process, with stage 1 being selection of schools randomly from Kandy District (an administrative unit) in Sri Lanka and stage 2 being selection of subjects randomly from the selected schools. A formal informed consent in writing was obtained from all participants who were 18 years of age or above and from parents or guardians of the participants who were below 18 years. All participants were in the age group of 16–19 years. The test group was diagnosed patients by a neurologist with migraine according to the International Headache Society (IHS) criteria,3rd edition (beta version) [1], with verification that they had more than 3 headache attacks but not more than 15 attacks per month. All the test patients underwent the procedure while they were having an acute manganous episode with headache and all were taken within 6 h from the onset of headache. None of them had taken acute treatment for migraine before the procedure. The control group was age and sex matched healthy participants. Exclusion criteria considered were similar to previous study [11]; history of intracranial lesion or tumor, recent nasal or sinus infection, acute or chronic sinusitis, evidence of another infection (i.e., acute otitis media or pneumonia), history of allergic rhinitis, asthma or an underlying immune deficiency, cystic fibrosis, immotile cilia syndrome, recent head and facial trauma, runny nose, smoking, alcohol or drug abuse. Participants who were on hormonal therapy for any condition or illness, patients with psychiatric illness, patients on non–medical/non–nutritional treatment for migraine prevention such as acupuncture or psychotherapy, patients on fasting and had exercise or used any nasal drops or steam inhalation 1 h before the procedure and patients who did not consent.

This study was carried out as an outpatient study. All participants were studied only once. Exhaled Nitric Oxide and exhaled CO levels of participants were measured before the paranasal air auction and participants with high exhaled Nitric Oxide and exhaled CO levels were excluded to prevent contamination of NO and CO in lung air with paranasal air. Three normal oral breathings were taken before the breath test for NO and CO to wash out para nasal gases. Then, after maximal oral inspiration, subjects were instructed to exhale into the device mouthpiece of the NO and CO analyzer. Finally, the level of exhaled NO and oral CO content in exhaled air were recorded by the analyzers. Normal value of Fraction of Exhaled Nitric Oxide (FeNO) for Tunisian and Arab of adults of any age and height was 5- 26 ppb [12] and 5-17 ppb For North African, Arab children of any age [13]. Thus, 5-10 ppb was assumed and taken as the normal exhaled NO for adolescents in our study, as there is no reference range for Sri Lankan adolescents of any age. Exhaled CO levels in healthy adolescents are 1.01 +/− 0.12 ppm and was taken as a reference value in our study [14]. In out study sample none of the participants had high exhaled NO or exhaled CO.

During the nasal - paranasal air suction process, nasal and paranasal sinus air were sucked three consecutive times from each nostril. Each suction was for 10–second duration with a 10–second suction free period between two suctions. Thus, each subject was subjected to 60–second suction altogether. Test participants hold the breath by closing both nostrils by his or her own hand. Then they were instructed to open one nostril for the suction for 10 s. After 10 s suction free period, they were asked to close the opened nostril and open the other nostril for air suction from that nostril for 10 s.

With each suction, the level of nasal - paransal sinus NO and nasal - paranasal sinus CO in the out flow of the paranasal air were measured using the NO and CO analyzer. The NO and CO measurements from 3 suctions of each nostril were separately pooled and the average was considered as the measurement of each gas of each nostril in the statistical analysis. It was important to measure NO at a flow rate of 50 mL/s with subjects inhaling NO-free air [15]. Therefore the suction apparatus was adjusted to deliver the airflow rate at 50 mL/s to the analyzer from nasal outer orifices. As we did not have one unit gas analyzer to detect the level of both gases, the suction connector tube was connected to both NO and CO analyzers on the same pathway. Possible errors due to the connector were expected to be nullified as controls were also measured in the same way.

Even though this method did not measure paranasal air directly, we assumed that syphon action created by the high airflow suction, sucked out the paranasal air. This was more convenient than invasive direct methods to measure paranasal air. During the suction process the contralateral nostril was closed to increase the pressure difference between sinus cavity and nasal cavity so that sinus air could be sucked out. In addition, with this closure, external air via opposite nasal orifice being sucked could also be prevented. However, when sucking from one side nostril, NO from other side sinuses and oral air from environment can be also be sucked due to the induced negative pressure by the air suction. Air sucked out from the lung was minimized by giving instruction to the participant to keep the mouth open with closed glottis during the paranasal air suction.

### NO measurement

NO is measured by the analyzer using an electrochemical sensor, which is especially designed to meet the breath test standards defined by The European Respiratory Society (ERS) and American Thoracic Society (ATS) recommendation and the test guideline described in the ERS Task Force Report [15]. We used commercially available fractional exhaled nitric oxide (FeNO) analyzer: The NObreath® FeNO monitor, Bedfont Scientific Limited. This equipment is sensitive to NO concentration from 5 to 300 ppb and gave continuous recordings with a resolution of about 5 ppb with a response time below 10 s. As the analyzer needed the temperature to be below 30 °C and relative humidity within 10–80% range. So the testing times were arranged from 8 am to 10 am in a closed classroom, where the needed temperature and the humidity existed. In order to ensure the sample was taken at a constant flow rate, the monitor was held upright at all times during testing. Moreover, during the testing period, mobile phones and radio waves emitting devices were kept off in and around the testing site. Periodic flushing of the tube and the sampling system of the analyzer were done by passing dry air to remove moisture, which can have interference on NO measurements.

### Co measurement

The Micro Smokerlyzer® CO monitor of Bedfont Scientific Limited was used to measure the CO concentration of the sucked air. The mechanism of measuring CO by the analyzer is same as for measuring NO. The equipment is sensitive to CO concentration from 0 to 150 ppm and gave continuous recordings with a resolution of 1 ppm with a response time of below 17 s. Since testing of both NO and CO were carried out simultaneously, CO measurements reading were also made at a temperature below 30 °C and within 10-80% relative humidity. Moreover, CO testing also took place without any interference from radio waves. The analyzer needed the exhalation air with a low positive pressure 5–20 cm H_2_O, which was maintained by adjusting the nasal air suction and the same stable flow rate of 50 ± 5 mL/s was maintained.

### Other measurements

Apart from separate NO and CO measurements from paranasal air sucked from each of the two sides, other measurements made were, nasal air flow rate of each side, general headache pain before and after suction, and left–side and right–side tenderness (supraorbital) before and after suction. The severity of the headache was measured using a standard pain rating scale (0 being pain free and 10 being severe pain) before and after the air suction procedure. Supraorbital tenderness was assessed by the same examiner applying pressure over the supraorbital notch (lying between the nasion and the trochlea), where the supraorbital branch lies, until some blanching of their fingernail was discernible [16]. This was assessed on both right and left. The severity of tenderness felt to the subjects was measured using the same pain rating scale. The nasal air flow rates of left and right nostrils were also assessed by the same examiner by feeling and assigning a score according to a four-point scale of 0 to 3, representing the levels in the following order: no flow, mild flow rate, moderate flow rate and normal flow rate.

### Sample size

According to the study objectives, the key measurement was the NO level. Thus, the sample size was calculated to detect a mean difference of 100 ppm between the two groups with type I error rate of 0.05 and power of the test of 0.9. Based on prior information of standard deviation of 90 ppm, the sample size was calculated using PROC POWER of SAS University Edition as 19 per group. With a potential 10% dropout, 21 for each group were considered. However, out of those who gave the consent, there were only 20 patients and 22 healthy participants satisfying inclusion and exclusion criteria. Thus, all 42 were recruited to the study and there were no dropouts.

### Statistical analysis

Exploratory analysis of the data revealed that almost all response variables did not distribute normally. Thus, non–parametric methods were used for statistical analysis. Wilcoxon rank sum test was used to compare the population median between patient group and the control group with respect to each response variable. In addition, interaction between gender and group was studied for each response variable using the method suggested by Akritas et al. [17]. Positive and negative predictive values of NO were calculated as a diagnostic screening test. Correlation between NO levels and nasal airflow rates were studied using Spearman’s correlation test. The receiver-operating characteristic (ROC) curve of paranasal NO and CO levels of left and right sides, exhaled NO, and exhaled CO levels were developed to study effectiveness of them as tests for the diagnosis of migraine. Comparisons of ROC curves were done to identify most effective measurement in diagnosis of migraine. Statistical analysis was performed using SAS University Edition.

## Results

The summary of the results of the tests for normality of the response variables are presented in Table 1. According to the results of all four tests for normality, except for exhaled NO, for all the other response variables normal distribution is not indicated (*P* <  0.01). Hence, statistical analyses were performed using non–parametric methods.

**Table 1:** Normality test statistics and their *P* values for response variables

Test	Nasal air flow (L)	Nasal air flow (R)	NO (L)	NO (R)	CO (L)	CO (R)	Exhaled NO	Exhaled CO	Tenderness (L) before suction	Tenderness (L) after suction	Tenderness (R) before suction	Tenderness (R) after suction	General Headache before suction	General Headache after suction
Shapiro–Wilk	0.8726 (*P* < 0.01)	0.8475 (*P* < 0.01)	0.8047 (*P* < 0.01)	0.7473 (*P* < 0.01)	0.7120 (*P* < 0.01)	0.7528 (*P* < 0.01)	0.9470 (*P* = 0.05)	0.4107 (*P* < 0.01)	0.8253 (*P* < 0.01)	0.8644 (*P* < 0.01)	0.4884 (*P* < 0.01)	0.7570 (*P* < 0.01)	0.796 (*P* < 0.01)5	0.7980 (*P* < 0.01)
Kolmogorov–Smirnov	0.2558 (*P* < 0.01)	0.2487 (*P* < 0.01)	0.2361 (*P* < 0.01)	0.2566 (*P* < 0.01)	0.3546 (*P* < 0.01)	0.3308 (*P* < 0.01)	0.1149 (*P* = 0.15)	0.4498 (*P* < 0.01)	0.1906 (*P* = 0.06)	0.2732 (*P* < 0.01)	0.4520 (*P* < 0.01)	0.3755 (*P* < 0.01)	0.3319 (*P* < 0.01)	0.2885 (*P* < 0.01)
Cramer–von Mises	0.4120 (*P* < 0.01)	0.4441 (P*P* < 0.01)	0.4963 (*P* < 0.01)	0.4572 (*P* < 0.01)	0.8670 (*P* < 0.01)	0.7601 (*P* < 0.01)	0.1029 (*P* = 0.10)	2.0197 (*P* < 0.01)	0.1723 (*P* = 0.01)	0.2590 (*P* < 0.01)	0.9330 (*P* < 0.01)	0.4876 (*P* < 0.01)	0.4146 (*P* < 0.01)	0.3464 (*P* < 0.01)
Anderson–Darling	2.2138 (*P* < 0.01)	2.5461 (*P* < 0.01)	2.8745 (*P* < 0.01)	2.8681 (*P* < 0.01)	5.2708 (*P* < 0.01)	4.4358 (*P* < 0.01)	0.71250 (*P* < 0.01)	9.9064 (*P* < 0.01)	1.2480 (*P* < 0.01)	1.3202 (*P* < 0.01)	4.5063 (*P* < 0.01)	2.5093 (*P* < 0.01)	2.0666 (*P* < 0.01)	1.8707 (*P* < 0.01)
N	41	42	42	42	42	42	42	42	20	20	20	20	20	20

*L* right, *R* Right, *NO* Nitric Oxide, *CO* Carbon monoxid

Wilcoxon rank sum test results of the comparison between patient group and the control group are given in the Table 2. The *P* < 0.02 for all variables in Table 2 clearly indicate that there is a difference between the two groups with respect to all variables. According to Table 2, average ranks for flow rate of both nostrils are substantially low in patients compared to the control and it signifies the lower flow rate in patients compared to the controls. Of course, patient group nasal air flow (L) estimated median score of 2 was higher than estimated median score of 1 for the control group and nasal air flow (R) estimated median score of 2 was the same for both groups. However, there were higher values in the control group compared to the patient group for both nasal air flow (R) and nasal air flow (L) and thereby mean score (average rank) were higher for the control group. Table 2 shows that paranasal NO and CO concentrations, exhaled NO and exhaled CO are much higher (*P* <  0.01) in migraine patients than in controls. Paranasal median NO levels of left and right sides in the patient group were 132.5 ppb and 154 ppb respectively compared to corresponding values of 32 ppb and 34.5 ppb respectively in the control group. This shows high paranasal NO content is strongly associated with the presence of migraine. The pattern was same with CO content too (Table 2). In general, the difference between patients and the controls for paranasal NO and CO was in line with difference between patients and the controls for exhaled NO and exhaled CO. However, in patients, median exhaled was 7 ppb and it was relatively low compared to paranasal median NO values. In fact, it was the case with the control group too (Table 2). Moreover, there was no interaction (*P* > 0.19) between group and gender with respect to the five variables (Table 3). In other words, the difference between these two groups was consistent across both gender groups. Here, the interaction between group and gender could not be studied for both left and right CO and exhaled CO due to the fact that for both males and female of controls CO values were less than 1 ppb and recorded as exactly 1 ppb. With a constant value interaction effect cannot be estimated.

**Table 2:** Wilcoxon Rank Sum Exact Test results for patient control difference: Mean score and (median) for the two groups and P for the difference

Group	N	Nasal air flow (L)	Nasal air flow (R)	NO (L)	NO (R)	CO (L)	CO (R)	Exhaled NO	Exhaled CO
Patient	20	16.78 (2)	16.80 (2)	32.50 (132.5)	32.50 (154)	31.40 (2)	31.95 (2)	29.75 (7)	25.90 (1)
Control	22	25.02 (1)	25.77 (2)	11.50 (36)	11.50 (34.5)	12.50 (1)	12.00 (1)	14.00 (4)	17.50 (1)
*P*		0.01	0.02	< 0.01	< 0.01	< 0.01	< 0.01	< 0.01	< 0.01

*L* right, *R* Right, *NO* Nitric Oxide, *CO* Carbon monoxide

**Table 3:** F statistics of type 3 non–parametric tests for Group and Sex effect, and their interaction for response variables

Group	df	Nasal air flow (L)	Nasal air flow (R)	NO (L)	NO (R)	Exhaled NO
Group	1	6.02 (*P* = 0.01)	7.05 (*P* = 0.01)	118.31 (*P* < 0.01)	117.99 (*P* < 0.01)	28.48 (*P* < 0.01)
Sex	1	0.20 (*P* = 0.65)	0.61 (*P* = 0.44)	0.47 (*P* = 0.50)	0.79 (*P* = 0.38)	0.01 (*P* = 0.94)
Interaction between group and gender	1	0.37 (*P* < 0.54)	0.19 (*P* = 0.67)	0.76 (*P* = 0.39)	0.26 (*P* = 0.61)	0.13 (*P* = 0.72)

*L* right, *R* Right, *NO* Nitric Oxide

Results of analysis of tenderness and headache pain score difference before and after the suction of migraine patients are presented in Table 4. The *P* <  0.01 of both sign test and sign rank test shows that there is a significant difference in pain scores of all three pain types before and after suction. From the Table 4, it can be seen that tenderness median score has decreased from 7 to 2 and 8 to 2 in left and right sides respectively. In addition, general headache pain median has decreased from 6 to 2.

**Table 4:** Test statistics and *P* values for (before – after) score differences, and sample medians of patients

Test	Tenderness (L)	Tenderness (R)	Headache Pain
Sign test	10 (*P* < 0.01)	9.0 (*P* < 0.01)	10 (*P* < 0.01)
Sign rank test	105 (*P* < 0.01)	103.5 (*P* < 0.01)	105 (*P* < 0.01)
Median (before)	7	8	6
Median (after)	2	2	2
N	20	20	20

*L* right, *R* Right

The Spearman correlation analysis of eight gas measurements is given in Table 5. Estimated correlation coefficients indicate that patterns of relationship are consistent between left–side and right–side measurements. Moreover, the outcome from correlation is consistent with results reported in Table 3 that NO and CO concentrations are negatively correlated with the airflow rate. In addition, NO and CO concentrations are positively correlated indicating when one gas occurs in higher concentration, the other gas follows the same trend. However, there was no significant correlation between left and right nasal flow as well as between nasal flow and exhaled NO and exhaled CO.

**Table 5:** Spearman Correlation Coefficients between response variables

	Nasal air Flow (L)	Nasal air Flow (R)	NO (L)	CO (L)	NO (R)	CO (R)	Exhaled NO	Exhaled CO
Nasal air Flow (L)	1.00	−0.03	−0.47	−0.29	−0.46	−0.44	−0.03	−0.17
		0.85	<0.00	0.07	<0.01	<0.01	0.88	0.28
	41	41	41	41	41	41	41	41
Nasal air Flow (R)	−0.03	1.00	− 0.35	− 0.34	− 0.35	− 0.38	− 0.27	− 0.03
	0.84		0.03	0.03	0.02	0.01	0.08	0.84
	41	42	42	42	42	42	42	42
NO (L)	−0.47	−0.35	1.00	0.74	0.87	0.81	0.53	0.35
	<0.00	0.03		<0.01	<0.01	<0.01	<0.01	0.02
	41	42	42	42	42	42	42	42
CO (L)	−0.29	−0.34	0.74	1.00	0.74	0.82	0.68	0.59
	0.07	0.03	<0.01		<0.01	<0.01	<0.01	<0.01
	41	42	42	42	42	42	42	42
NO (R)	−0.46	−0.35	0.87	0.74	1.00	0.86	0.37	0.37
	<0.01	0.02	<0.01	<0.01		<0.01	0.01	0.02
	41	42	42	42	42	42	42	42
CO (R)	−0.44	−0.38	0.81	0.82	0.86	1.00	0.54	0.49
	<0.01	0.01	<0.01	<0.01	<0.01		<0.01	<0.01
	41	42	42	42	42	42	42	42
Oral NO	−0.03	−0.27	0.53	0.68	0.37	0.54	1.00	0.44
	0.88	0.08	<0.01	<0.01	0.01	<0.01		<0.01
	41	42	42	42	42	42	42	42
Oral CO	−0.17	−0.03	0.35	0.59	0.37	0.49	0.44	1.00
	0.28	0.84	0.02	<0.01	0.02	<0.01	<0.01	
	41	42	42	42	42	42	42	42

Note: In each cell, entries in order are (i) Spearman *r*, (ii) Prob > |*r*| under H0: Rho = 0, and (iii) N

*L* right, *R* Right, *NO* Nitric Oxide, *CO* Carbon monoxide

**Fig. 1:**
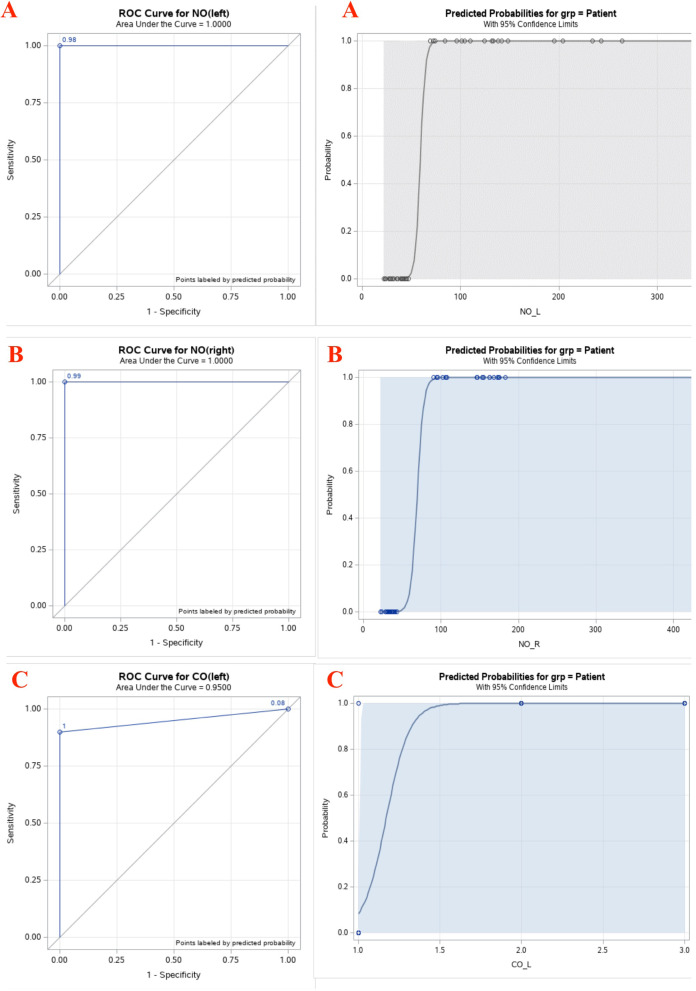
**A** = The receiver characteristic operating (ROC) curve and predicted probabilities for NO (Left). **B** = The receiver characteristic operating (ROC) curve and predicted probabilities for NO (Right). **C** = The receiver characteristic operating (ROC) curve and predicted probabilities for CO (Left)

**Fig. 2:**
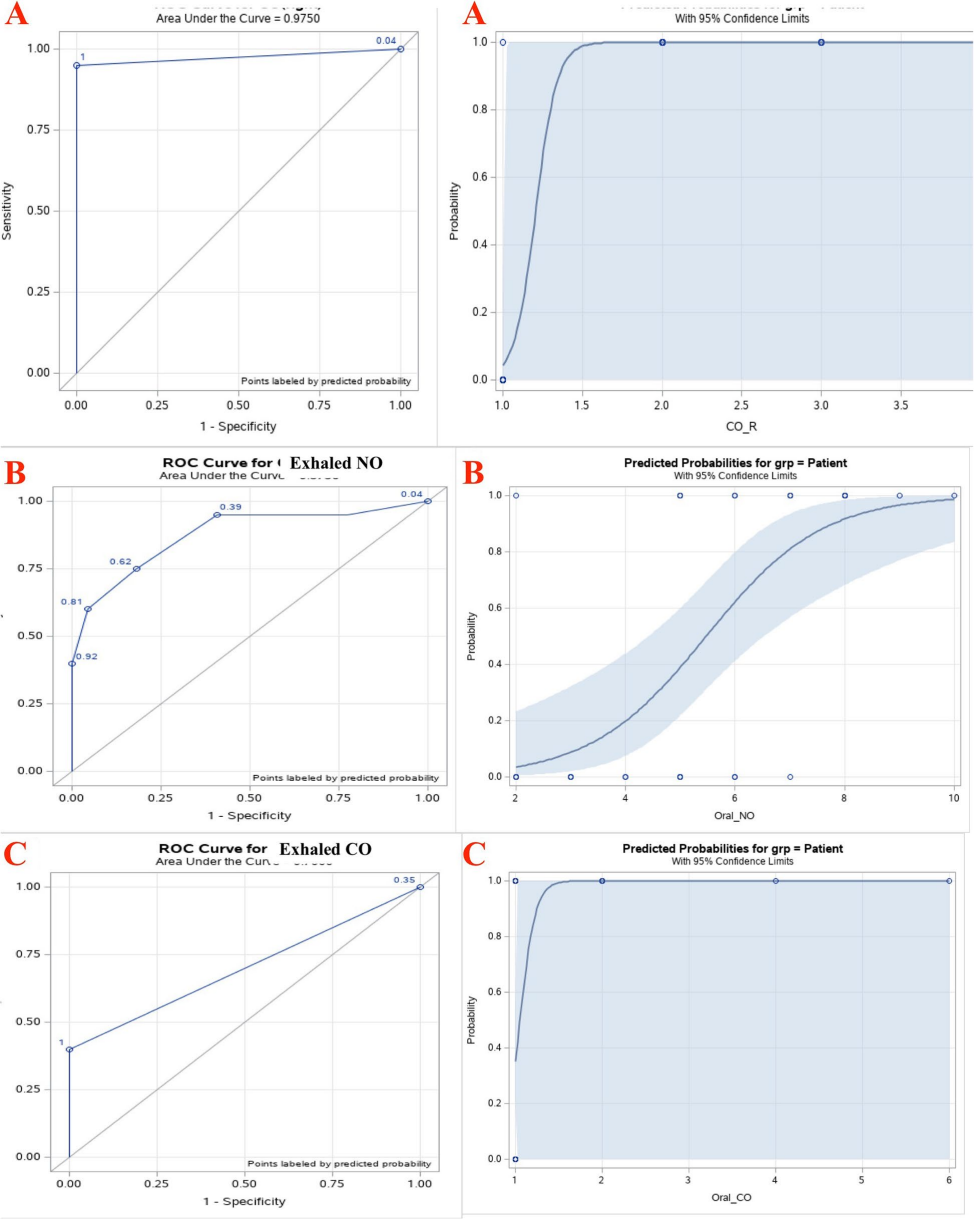
**A** = The receiver characteristic operating (ROC) curve and predicted probabilities for CO (Right). **B** = The receiver characteristic operating (ROC) curve and predicted probabilities for exhaled NO. **C** = The receiver characteristic operating (ROC) curve and predicted probabilities for exhaled CO

In order to investigate the possibility of using paranasal NO and CO as a diagnostic tool of migraine, sensitivity and specificity analysis were performed for those variables. With these two variables it was very easy to identify cut off points. With para nasal sinus NO, all patients had values above 50 ppb and controls had values below 50 ppb. With paranasal sinus CO, all patients had values 1 ppm or above, but all controls had this value less than 1 ppm but recorded as 1 ppm. Thus, the cut off values considered for NO and CO were above 50 ppb and above 1 ppm respectively. The cross tabulation of true diagnosis and diagnosis based on NO (L) with 50 ppb as the cutoff point is given in Table 6, and the sensitivity, specificity, positive predictive value (PPV) and negative predictive value (NPV) based on Table 6 are given in Table 7. By looking at Tables 6 and 7 it can be clearly seen that perfect classification of 100% sensitivity, specificity, PPV and NPV can be done based on NO at 50 ppb. In fact, this was same for NO(R) (Tables 8 and 9). The reason for perfect classification is due to the fact that there was no overlap between NO ranges of patient and control groups. Output from similar analysis based on CO with a cut off value of above 1 ppm is given in Tables 10 and 11, 12 and 13. From those tables, it could be seen that almost perfect classification can be made based on CO with 1 ppm too. In fact, by means of CO perfect classification can be made for specificity and PPV but almost perfect classification can be made for sensitivity and NPV. Cross tabulation with respect to exhaled based on the cutoff point 6 ppb and above, and corresponding sensitivity, specificity, PPV and NPV are given in Tables 14 and 15. Similarly, cross tabulation with respect to Exhaled CO based on the cutoff point above 1 ppm, and corresponding sensitivity, specificity, PPV and NPV are given in Tables 16 and 17. According to Tables 14, 15, 16, and 17, it can be seen that using them also a good classification can be made but classification from these two is not as prominent as the classifications based on paranasal NO and CO. Note that all cutoff points were decided based on the predicted probability plots of Figs. 1 and 2.

**Table 6:** Cross tabulation of NO (L) by group

NO(L)	Group	
	Patient	Control
> 50	20	0
≤ 50	0	22

Fisher’s exact test statistic F = 20, *P* < 0.0001

**Table 7:** Sensitivity and Specificity of NO (L)

Statistic	Estimate	Standard Error	95% Confidence Limits	
Sensitivity	1.0000	0.0000	1.0000	1.0000
Specificity	1.0000	0.0000	1.0000	1.0000
Positive Predictive Value	1.0000	0.0000	1.0000	1.0000
Negative Predictive Value	1.0000	0.0000	1.0000	1.0000

**Table 8:** Cross tabulation of NO(R) by group

NO(R)	Group	
	Patient	Control
> 50	20	0
≤ 50	0	22

Fisher’s exact test statistic F = 20, *P* < 0.0001

**Table 9:** Sensitivity and Specificity of NO(R)

Statistic	Estimate	Standard Error	95% Confidence Limits	
Sensitivity	1.0000	0.0000	1.0000	1.0000
Specificity	1.0000	0.0000	1.0000	1.0000
Positive Predictive Value	1.0000	0.0000	1.0000	1.0000
Negative Predictive Value	1.0000	0.0000	1.0000	1.0000

**Table 10:** Crosstabulation of CO(L) by group

CO(L)	Group	
	Patient	Control
> 1	18	0
≤ 1	2	22

Fisher’s exact test statistic F = 18, *P* < 0.0001

**Table 11:** Sensitivity and Specificity of CO(L)

Statistic	Estimate	Standard Error	95% Confidence Limits	
Sensitivity	0.9000	0.0671	0.7685	1.0000
Specificity	1.0000	0.0000	1.0000	1.0000
Positive Predictive Value	1.0000	0.0000	1.0000	1.0000
Negative Predictive Value	0.9167	0.0564	0.8061	1.0000

**Table 12:** Crosstabulation of CO(R) by group

CO(L)	Group	
	Patient	Control
>1	19	0
≤1	1	22

Fisher’s exact test statistic F = 19, *P* < 0.0001

**Table 13:** Sensitivity and Specificity of CO(R)

Statistic	Estimate	Standard Error	95% Confidence Limits	
Sensitivity	0.9500	0.0487	0.8545	1.0000
Specificity	1.0000	0.0000	1.0000	1.0000
Positive Predictive Value	1.0000	0.0000	1.0000	1.0000
Negative Predictive Value	0.9565	0.0425	0.8732	1.0000

**Table 14:** Cross tabulation of Exhaled NO by group

Oral NO	Group	
	Patient	Control
≥ 6	15	4
< 6	5	18

Fisher’s exact test statistic F = 15, *P* = 0.0005

**Table 15:** Sensitivity and Specificity of Exhaled NO

Sensitivity and Specificity				
Statistic	Estimate	Standard Error	95% Confidence Limits	
Sensitivity	0.7500	0.0968	0.5602	0.9398
Specificity	0.8182	0.0822	0.6570	0.9794
Positive Predictive Value	0.7895	0.0935	0.6062	0.9728
Negative Predictive Value	0.7826	0.0860	0.6140	0.9512

**Table 16:** Cross tabulation of exhaled CO by group

Oral CO	Group	
	Patient	Control
> 1	8	0
≤ 1	12	22

Fisher’s exact test statistic F = 8, *P* < 0.0011

**Table 17:** Sensitivity and Specificity of exhaled CO

Sensitivity and Specificity				
Statistic	Estimate	Standard Error	95% Confidence Limits	
Sensitivity	0.4000	0.1095	0.1853	0.6147
Specificity	1.0000	0.0000	1.0000	1.0000
Positive Predictive Value	1.0000	0.0000	1.0000	1.0000
Negative Predictive Value	0.6471	0.0820	0.4864	0.8077

The receiver characteristic operating (ROC) curve and predicted probabilities for the patient group based on NO and CO are given in Figures 1 and 2. The areas under ROC curves for NO(L) and NO(R) were unity and it is the maximum possible value under a ROC curve (Fig. 1A and B). Similarly, the area under the ROC of CO (L) and CO(R) were 0.95 and 0.975 respectively (Figs. 1 and 2C and A). In addition, the probability for the patient group graphs of NO (L) and NO (R) show that when NO value is higher than 50 ppb, predicted probability of a subject to be a patient is unity and when the value lower than 50 this probability is zero. Based on both CO (L) and CO(R), with a value above 1.5 ppm, predicted probability of a subject to be a patient is unity. However, when the CO concentration value is less than 1.5 ppm, probability of a subject to be a patient is between 0 and 1, but not exactly equal to zero as in the case with NO. With exhaled NO and exhaled CO, the area under ROC curves were 0.875 and 0.7 respectively (Fig. 2B and C). Thus, these two values are slightly smaller compared to the corresponding area values for paranasal NO and CO. From the probability plots of Fig. 2B and C it can be seen higher probabilities are indicated above the cutoff points, but those probability values were not close to zero and unity from the two directions, as compared to the case with paranasal NO.

**Fig. 3:**
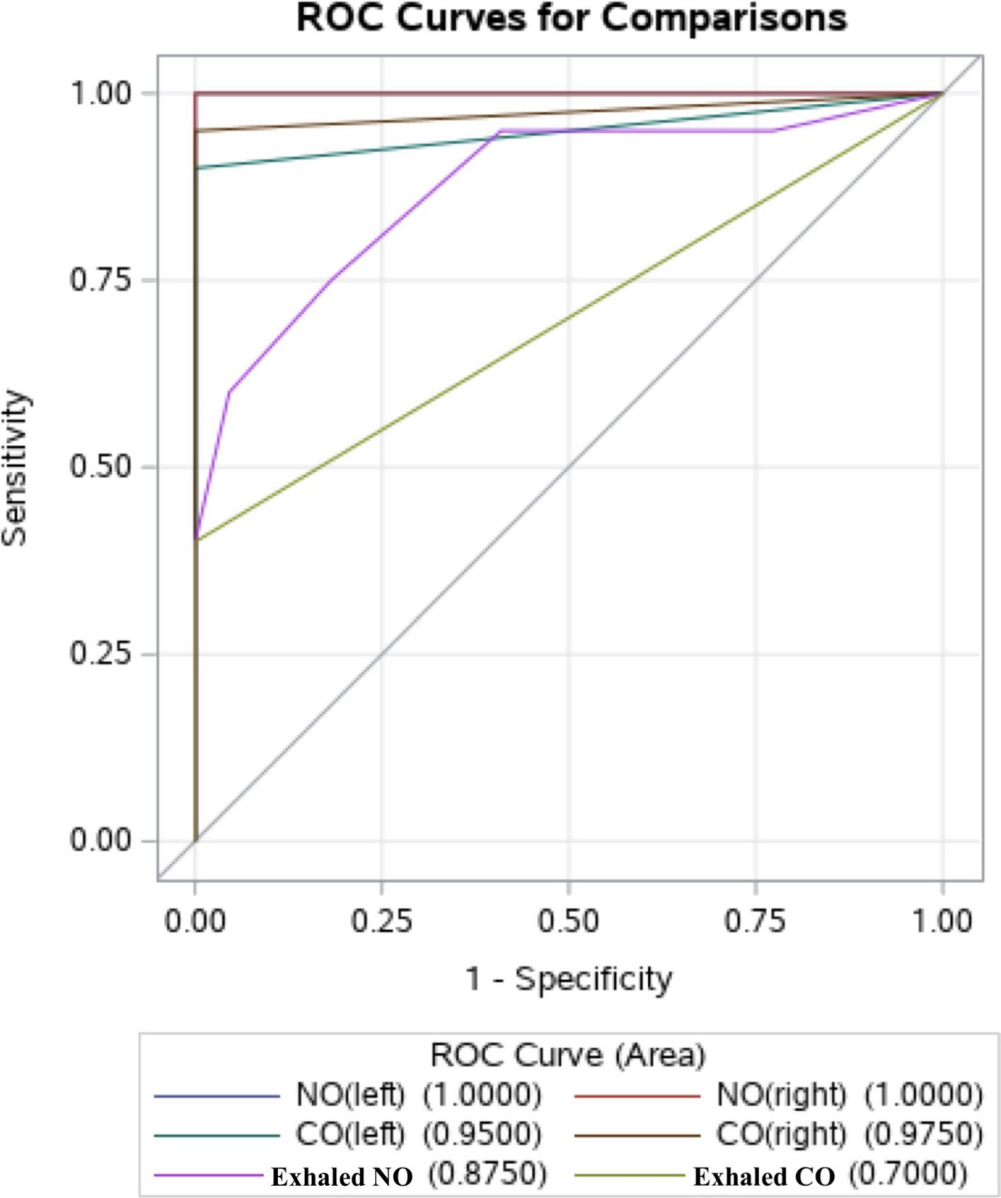
The relative positions of the estimated ROC curves for 6 tests (variables)

The Fig. 3 shows the relative positions of the estimated ROC curves for 6 tests (variables). According to the plot it is clear all curves lie above the 0.5 line and all 6 tests can be used for classification of patients and controls. The estimated ROC area values, their standard errors, and 95% confidence interval for are presented in Table 18. No interval contains the value 0.5 and thus, it indicates all those tests have the potential of classifying patients and controls. Comparison of 6 ROC curves were done by means of contrasts and those results are presented in Tables 19 and 20 tables. The *P* <  0.0001 for chi–square of the contrast analysis (Table 19) indicated that at least two curves are different with respect to area under the curve. Since exhaled CO had the least sensitivity out of 6 tests, using the exhaled CO ROC curve as the reference curve other curves were evaluated. Results of the evaluation are given in Table 20 and *P* values indicate that other tests are better than oral CO test (*P* = 0.01). However, the confidence interval for paranasal NO (both left and right) and paranasal CO (both left and right) contains the maximum value unity and thus those four tests are better than the other two. Out of those four, the confidence limits for paranasal NO (both left and right) contains only the value unity and thereby emphasizes a perfect classification using those two tests.

**Table 18:** ROC association statistics

ROC Model	Mann-Whitney			
	Area	Standard Error	95% Wald Confidence Limits	
NO (left)	1.0000	0	1.0000	1.0000
NO (right)	1.0000	0	1.0000	1.0000
CO (left)	0.9500	0.0344	0.8826	1.0000
CO (right)	0.9750	0.0250	0.9260	1.0000
Exhaled NO	0.8750	0.0552	0.7668	0.9832
Exhaled CO	0.7000	0.0562	0.5899	0.8101

**Table 19:** ROC Contrast Test Results

Contrast	DF	Chi-Square	Pr > ChiSq
Reference = CO (left)	4	37.5785	<.0001

**Table 20:** ROC Contrast Estimation and Testing Results by Row

Contrast	Estimate	Standard Error	95% Wald Confidence Limits		Chi-Square	Pr > ChiSq
NO (left) - exhaled CO	0.3000	0.0562	0.1899	0.4101	28.5000	<.0001
NO (right) - exhaled CO	0.3000	0.0562	0.1899	0.4101	28.5000	<.0001
CO (left) - exhaled CO	0.2500	0.0574	0.1376	0.3624	19.0000	<.0001
CO (right) - exhaled CO	0.2750	0.0676	0.1425	0.4075	16.5396	<.0001
Exhaled NO - exhaled CO	0.1750	0.0708	0.0363	0.3137	6.1133	0.0134

## Discussion

We found a significant drop in migraine headache and supra orbital tenderness with paranasal air suction in our past study [11]. The P values of both sign test and sign rank test showed that there is a significant positive difference in pain scores before and after the air suction. In addition, the mean pain score drop after air suction was very high (*P* <  0.0001) in migraine patients compared to the controls. Hence, as in our past study, this study confirmed a significant drop in pain in migraine headache and supra orbital tenderness after the paranasal air suction.

This study demonstrates that subjects with migraine had median NO content of 132.5 ppb and 154 ppb on left and right sides in air sucked out from paranasal sinuses during an acute episode, respectively compared to 36 ppb and 34.5 ppb respectively in controls (*P* <  0.0001). These data strongly indicated that migraine patients in general have comparatively 4-5 times higher values of NO in paranasal air compared to controls. Moreover, CO concentration in paranasal air was also very high (*P* <  0.0001) in migraine patients than controls, where patients had a median CO level of 2 ppm (both left and right sides) compared to 1 ppm (both left and right sides) in controls. These results provide adequate evidence on the fact that paranasal air of migraine patients contains high level of NO and CO compared to healthy subjects, and thus it can be deduced that occurrence of high level of NO and CO can have a direct association with the presence of migraine headache. Further, these two gases with cutoff points 50 ppb for NO and 1 ppm for CO have shown very high sensitivity, specificity, PPN and NPV with migraine headache. Therefore, these two gas tests can clearly be used to distinguish migraine patents from healthy persons. ROC curve analysis also has confirmed the fact that paranasal NO and CO can be used as tests to diagnose migraine. In fact, ROC analysis has further indicated that paranasal NO is a perfect test to diagnose migraine. In fact, no specific laboratory or radiological test has been established for diagnosis of migraine headaches so far. However migraine remains a clinical diagnosis most of the time but it can be proposed that paranasal NO and CO are useful measurements during an acute episode in difficult circumstances such as functional headache disorders or when headache features overlap with others. Migraine can be diagnosed even during pain-free period by clinical interview, neurologist do investigations to exclude other causes for headache in some instances or on patient’s request. Among those investigations, X ray sinuses view, brain MRI, CT scan of brain and paranasal views, angiogram, EEG are quite common. However, according to this study, if clinicians can have facility to make a simple measure of nasal and paranasal NO, supportive evidence can be generated without using sophisticated equipment as well as avoiding unnecessary expose of patients to radiation. Another important finding of this study is that the investigation itself provides relief to acute migraine headache. This can also be used as a basis for future research for finding a new investigation for migraine.

Before coming to the conclusion of the causative molecules, basic physiological action of NO and CO and other research evidence must be understood and evaluated. Many vasoactive neuropeptides such as substance P, neurokinin A [18] calcitonin gene–related peptide [19], NO [3] and serotonin [20] have been hypothesized in migraine pathology. Nitroglycerin (GTN) could induce migraine-like headache via Nitric oxide synthetase activation and increased production of NO [21–23] and migraine sufferers were more sensitive to GTN than normal controls. GTN infusion induce a dose- dependent headache in all groups and in migraine sufferers the headache was more severe, longer lasting than normal volunteers [24]. Isosorbide mononitrate also produce prolong headache by delivering NO in a prolonged fashion [25]. NO in guinea-pig dura mater induced extravasation and other changes similar to those induced by neurogenic inflammation [26]. NO in guinea-pig dura mater induces extravasation and other changes similar to those induced by neurogenic inflammation [25]. NO is also involved in nociceptive processing in the central nervous system sensitisation of pain pathways in the spinal cord and Nitric oxide synthetase inhibition reduces central sensitisation [21]. Nitric oxide metabolites were significantly higher in maxillary sinuses of patients with chronic sinusitis so we can assume that NO has an important role in inflammation [27]. On the other hand, excess NO synthesized in the tissue is a pro inflammatory and pro apoptotic factor [28, 29]. Vitamin B12 (NO scavenger) was proposed to be a possible treatment option for chronic migraine management [30, 31] was a supportive evidence for sinus hypoxic nitric oxide induced migraine hypothesis. On the other hand CO is also an endogenously produced pain-modulating neurotransmitter and CO may play an important role in the mechanisms of migraine CO by inducing hypoxia, increasing nitric oxide signaling, activation of cyclic guanosine monophosphate pathways, cerebral vasodilation and production of free radicals [32]. Excess CO might contribute to stimulate peripheral trigeminal neuronal endings via vasodilator effect in the mucosa. Both NO and CO are neurotransmitters that interact as co–transmitters as well [33]. It was described that that NO may not act properly in the absence of CO generation in enteric mucosa and it was explained that CO may enhance n NOS catalytic activity or facilitate NO release from enteric neurons [33]. Smoking is a precipitating factor for migraine [34] and high levels of CO and NO were found in cigarette smokes [35]. Theses observation and findings support to our findings of association between CO and NO in migraine attack.

According to airflow rate analysis, migraine patients had low airflow rates and NO and CO concentrations negatively correlated with the airflow rates. So we can assume that migraine patients had low flow rates and hypoxia which may increase production and stagnation of NO and CO in paransal sinuses. The patients were studied during an episode of migraine and in migraine, there may be a partial or complete nasal obstruction of nostrils or ostial track due to parasympathetic over activity causing mucosal oedema. Studies have shown that the prevalence of migraine at high altitude is high [36] and it was assumed that hypoxia in high altitude may increase NO activity to cause headache [37]. So we can assume that ventilation of sinus cavity by mechanical interference could be a strategy to reduce hypoxia, nitric oxide production, absorption and stagnation.

One limitation of the study was that it was difficulty to calculate or measure the actual gases concentration in the sinuses. However, those measures are invasive, costly and difficult. We also measured NO and CO only. Lastly this study was done using adolescent of age 16-19 years and not the general adult population.

In conclusion, NO level of above 50 ppb was found in sinus air of all school adolescents during an acute migraine episode and this value is below 50 ppb in those did not have migraine. Similarly, CO level above 1 ppm was found in all migraine patients during an acute episode and those who did not have migraine had CO level up to 1 ppm. Thus, paransal NO and CO concentrations can be used to distinguish between individuals with and without migraine during an episode and especially NO with a cutoff value of 50 ppb can be used as a diagnostic test for migraine. Moreover, air suction relieves patients from migraine headache as well as supraorbital tenderness. It seems this relief can be achieved with about three consecutive 50 L /min speed suction of 30 s for each nostril. Increased sinus NO and CO during acute episode of migraine is an observation we had and acute migraine can be relieved by evacuation of vasoactive and neuroactive nasal and paranasal sinus NO and CO. We agree that further studies are needed to conclude that NO and CO can be a causative molecule for migraine headache.

## Data Availability

The datasets supporting the conclusions of this article are included within the article. If need of any other data or raw data contact the corresponding author via tharukaherath11@gmail.com and will be provided on request.

## Abbreviations

NO: Nitric Oxide
CO: Carbon Monoxide
R: Right
L: Left
ROC: Receiver characteristic operating
PPV: Positive predictive value
NPV: Negative predictive value
GTN: Nitroglycerin
